# Malnutrition‐related cardiomyopathy in a pediatric patient with autism spectrum disorder

**DOI:** 10.1002/jpr3.12142

**Published:** 2024-10-25

**Authors:** Alexandria Speakman, Ajay Kaul, Clifford Chin, Stavra Xanthakos, Marialena Mouzaki

**Affiliations:** ^1^ Department of Gastroenterology Cincinnati Children's Hospital Medical Center Cincinnati Ohio USA; ^2^ Department of Cardiology Cincinnati Children's Hospital Medical Center Cincinnati Ohio USA

**Keywords:** autism, heart failure, malnutrition, restrictive eating

## Abstract

We present a case of a 5‐year‐old male patient with history of autism spectrum disorder and chronic malnutrition secondary to an extremely restrictive diet who presented with life‐threatening anemia and heart failure secondary to malnutrition‐related cardiomyopathy. This case highlights the importance of recognizing nutritional deficiencies in high‐risk individuals and screening for nutritional deficiencies in at‐risk children. Furthermore, it underscores the need for early recognition and intervention as malnutrition‐related cardiomyopathy can have significant morbidity.

## 1 INTRODUCTION

Cardiomyopathy is characterized by impaired cardiac muscle function. Among the heterogeneous causes of cardiomyopathy, malnutrition‐related cardiomyopathy is a rare, but potentially life‐threatening condition. According to the Pediatric Cardiomyopathy Registry, 24% of children with dilated cardiomyopathy are malnourished.^1^ Proper cardiac function requires adequate energy, protein, and micronutrients such as carnitine, selenium, thiamine, and niacin.^2^, ^3^, ^4^, ^5^ Deficiencies in these nutrients can cause cardiac dysfunction, ranging from mild to heart failure (HF).^4^ Autism spectrum disorder (ASD) is characterized by impairments in social communication and the presence of restricted, repetitive behaviors. Individuals with ASD often have unique dietary preferences and sensory sensitivities that increase the risk of nutritional deficiencies.^6^ We present a case of malnutrition‐related cardiomyopathy in a child with ASD, highlighting the complex interplay between neurodevelopmental disorders, malnutrition, and cardiomyopathy.

## 2 CASE REPORT

A 5‐year‐old male with ASD and a long history of an extremely restrictive diet presented to the emergency department with dyspnea, fatigue, poor appetite, and difficulty performing daily activities. Over the week before presentation, caretakers reported increasing fatigue, irritability, and abdominal distention. Two days before presentation, he developed anorexia, with intake limited to water, and labored breathing. A review of the systems was negative for edema, rashes, vomiting, diarrhea, or fevers. Over the preceding 4 years, the patient's diet was limited to mango puree baby food, mango juice, whole milk, and water. He refused any dietary supplements or medications and had a history of constipation. There was no history of trauma, recent illness, or syncope. Family history was significant for siblings with ASD and restrictive eating habits.

**Figure 1:**
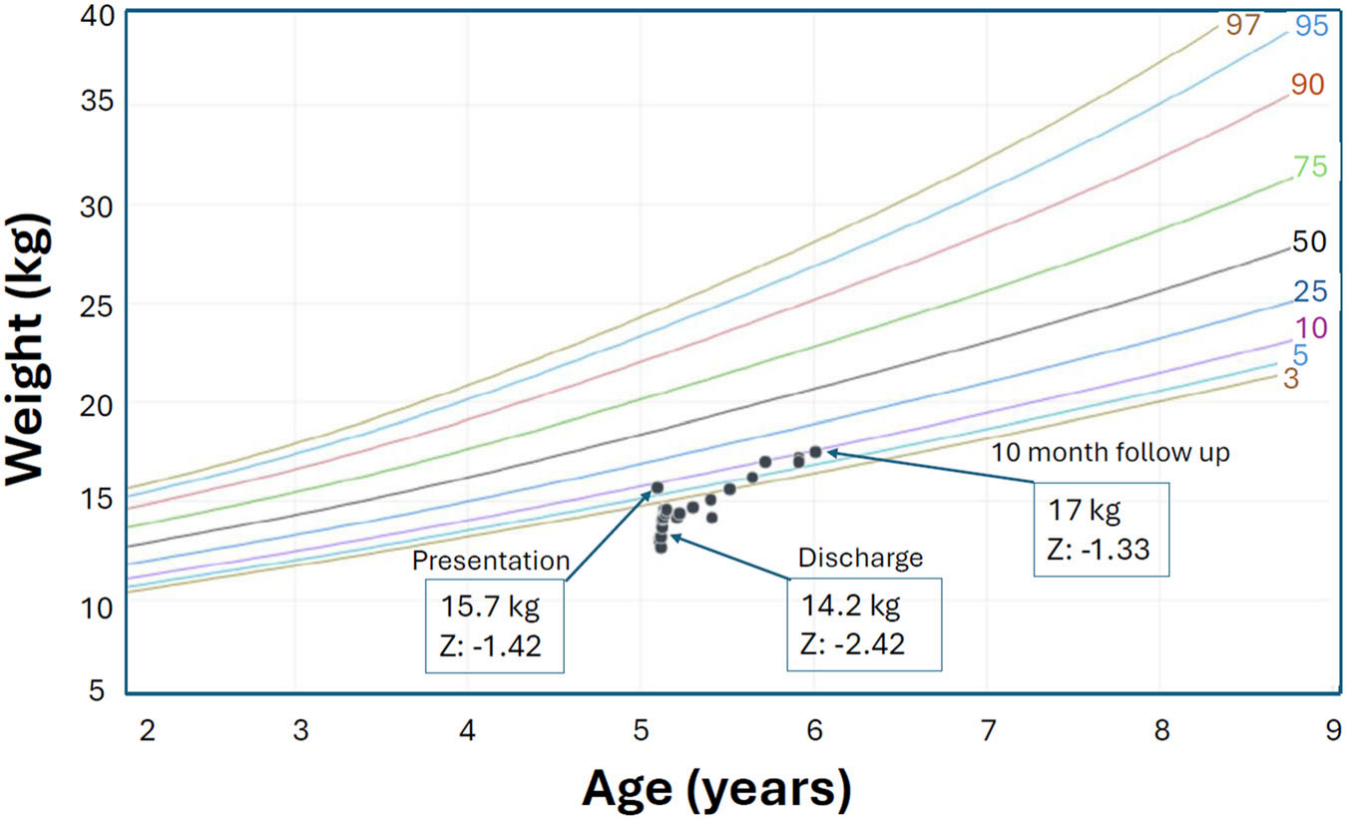
CDC growth chart with weights and *z*‐scores from presentation, discharge, and 10 months after discharge. CDC, Centers for Disease Control and Prevention.

**Figure 2:**
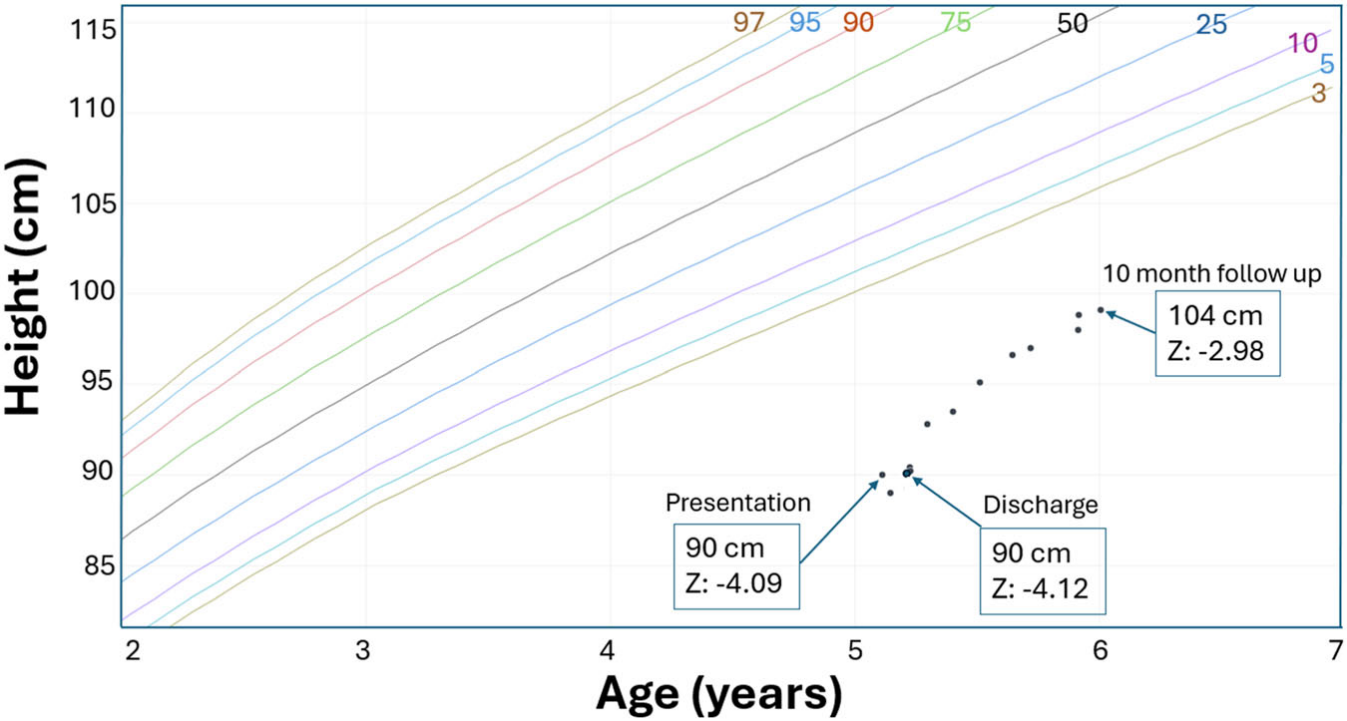
CDC growth chart with heights and *z*‐scores from presentation, discharge, and 10 months after discharge. CDC, Centers for Disease Control and Prevention.

**Figure 3:**
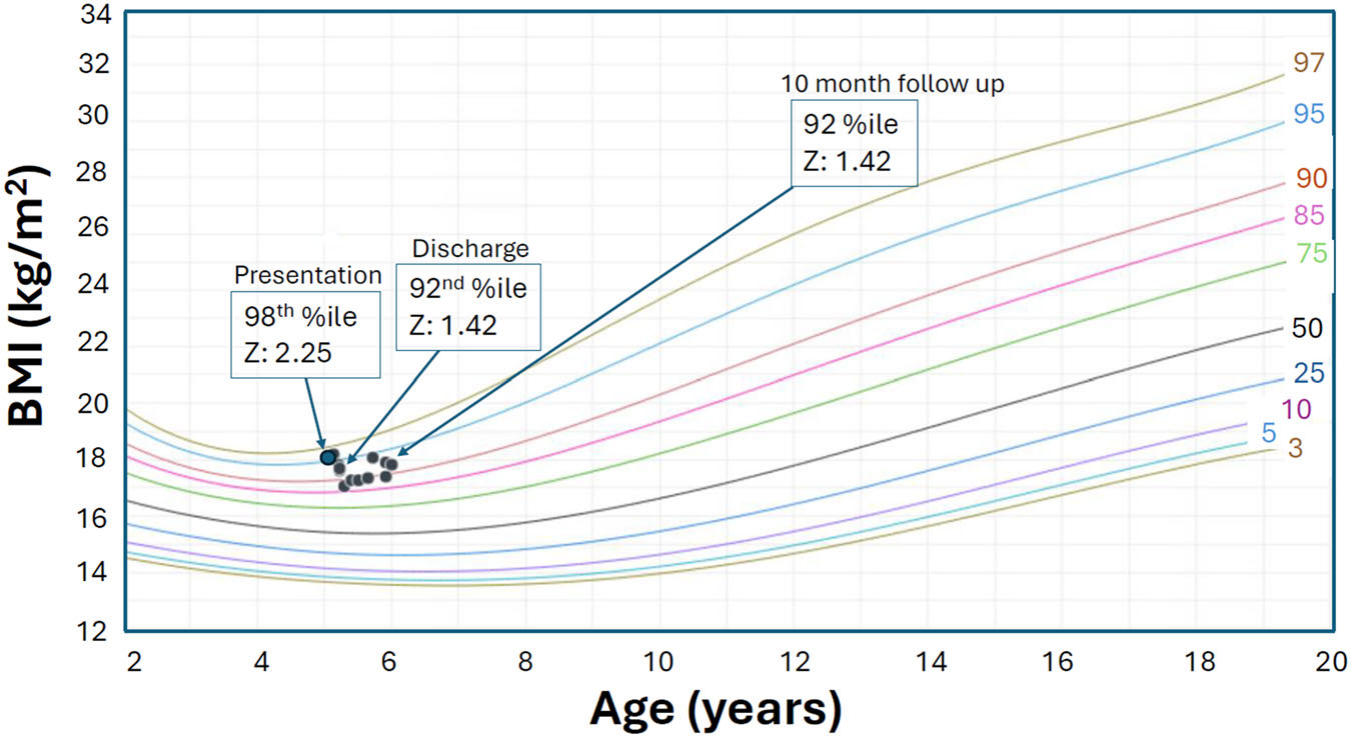
CDC growth chart for BMI with percentiles and *z*‐scores from presentation, discharge, and 10 months after discharge. BMI, body mass index; CDC, Centers for Disease Control and Prevention.

His initial vital signs were remarkable for tachycardia (~130 beats per minute) and tachypnea (~30 breaths per minute), with normal blood pressure and oxygen saturation. His anthropometrics revealed severe malnutrition with a mid‐upper arm circumference (MUAC) *Z*‐score of −3.15, and stunting (height of 90 cm, <0.01 percentile, *z*‐score of −4); his weight was 15.7 kg (7.8 percentile, *z*‐score of −1.4). Body mass index (BMI) *z*‐score was 2.25 (Figures 1, 2, 3). He appeared thin with pale loose skin, sarcopenic, with prominent clavicles, a Grade II/VI systolic heart murmur along the left sternal border, suprasternal and subcostal retractions and a non‐tender, moderately distended abdomen. He had no peripheral edema, temporal wasting, hair thinning, skin rashes, oral ulcers, or tongue abnormalities.

Initial laboratory investigations revealed a hemoglobin of 2.0 (normal 11.5–13.5) g/dL, mean corpuscular volume 66 (normal 75–87), ferritin of 4.7 (normal 10–150) ng/mL, and albumin of 2.6 (normal 3.5–4.7) g/dL. Chest and abdominal X‐rays demonstrated a moderately enlarged cardiac silhouette with associated splaying of the carina suggestive of cardiomegaly with left atrial enlargement. Initial echocardiogram demonstrated severe left ventricular dilation (*z*‐score = 7.7) with dysfunction (left ventricular ejection fraction [LVEF] = 29%) and a severely dilated left atrium.

After receiving packed red blood cell transfusions for the anemia, he developed transfusion‐associated circulatory overload requiring transfer to the cardiac intensive care unit. Treatments included a milrinone drip, sacubitril/valsartan, carvedilol, spironolactone, aspirin, and nutritional rehabilitation. Due to his tenuous status, he started exclusive parenteral nutrition (PN) on hospital day (HD) 2. He was initially prescribed 12 kcal/kg/day (25% of his resting energy expenditure) to avoid refeeding syndrome. His PN had additional thiamine (100 mg), selenium (2 mcg/kg), carnitine (20 mg/kg), and zinc (300 mcg/kg) due to presumptive deficiency from severe and chronic dietary restriction. By HD5, he was hemodynamically stable, the milrinone drip was discontinued, and he started enteral nutrition with a 1.0 kcal/mL polymeric formula via nasogastric tube (NGT), which he tolerated well (Figure S1). Oral‐motor feeding therapy was initiated, but as he refused all trials of preferred foods, further evaluation was limited.

After 14 days, he was discharged with an LVEF of 29%, a weight of 14.2 kg (0.78 percentile, *z*‐score −2.42—lower than on admission, presumably due to diuresis), a height of 90 cm (<0.01 percentile, *z*‐score −4) and BMI *z*‐score of 1.42. MUAC was not available at discharge. Whole‐exome sequencing and genetic microarray testing were completed due to short stature and unrevealing. This further supported the concern regarding malnutrition‐driven stunting. He was discharged with carvedilol, furosemide, and sacubitril/valsartan, as well as NGT feeding that provided 100% of his nutritional requirements. He continued his limited diet orally and was also given a pediatric multivitamin with iron. Ten months after discharge, the LVEF was 48%, weight 17.5 kg (9.3 percentile, *z*‐score −1.3), and height 99.1 cm, 0.07 percentile, *z*‐score −3.2). BMI *z*‐score (1.42) and hemoglobin (14 g/dL) were stable (no MUAC data collected at this visit). He continued HF medications and NGT feeds providing ~62 kcal/kg/day along with a pediatric multivitamin with iron and mango purees by mouth.

## 3 DISCUSSION

This case highlights the importance of recognizing nutritional deficiencies in children with restrictive eating, as they can be life‐threatening. Nutritional deficiencies that can cause HF include micronutrients (i.e., selenium, carnitine, thiamine, iron, and niacin)^4^, ^5^, ^7^ and protein deficiency.^8^ Anemia from chronic iron deficiency reduces oxygen delivery to tissues. A chronic compensatory increase in cardiac output and stroke volume in response to decreased oxygen delivery can lead to impaired heart function.^9^ Other nutrients are enzymatic co‐factors (thiamine, selenium, and carnitine) required for cellular energy transfer, which when absent can also impair cardiac function.^4^ Marasmus and kwashiorkor can also lead to cardiac dysfunction,^2^ though less common in the United States.

Food selectivity is a large concern for patients with ASD and increases the risk of nutritional deficiencies. While there are no standard screening tests to detect nutritional deficiencies, anthropometrics, biochemical markers (e.g., serologic levels of folate, Vit B6, Vit B12, Vit D, and others), and diet history (i.e., increased sensory issues, decreased food variety and micronutrient intake) can be used to identify those at risk.^10^ In patients with a highly selective diet and HF due to multiple nutrient deficiencies, supplementation with Coenzyme Q10 (CoQ10) and carnitine has improved cardiac function without any adverse effects.^4^ Prior research on micronutrient supplementation in cardiomyopathy and HF has mainly focused on single micronutrients (primarily CoQ10 and carnitine) with few studies investigating multiple‐nutrient supplementation.^4^ Information regarding dosing of micronutrients and duration of treatment is sparse and conflicting, warranting further research. Empiric treatment with thiamine, selenium, and carnitine in this patient was prescribed, as his restrictive diet was thought to have led to deficiencies in these micronutrients, which are found in whole grains, meats, nuts, and legumes. Serum levels of these micronutrients were not measured before supplementation. Thiamine levels can be inaccurate (which is why clinicians often rely on indirect markers of thiamine deficiency, namely lactate elevations, which in this case may have also been secondary to HF‐driven hypoperfusion).^11^ Selenium and carnitine levels may take several days to result. As such, empiric treatment is often initiated in patients thought to be at high risk for these deficiencies, to prevent further clinical deterioration.

Finally, linear stunting can result from chronic undernutrition and result in normal or paradoxically high BMI *z*‐score, as seen here. BMI is thus suboptimal to screen for nutritional deficiencies in chronically malnourished patients. MUAC is more sensitive to detect malnutrition and track nutritional status over time^12^ and is not typically affected by fluid shifts/hydration status even with edema/ascites^12^ which can occur with HF.^7^ However, both weight and MUAC may be stable in those who maintain adequate caloric intake, despite an otherwise restrictive diet.

## 4 CONCLUSION

Malnutrition‐related cardiomyopathy may have significant morbidity and requires early diagnosis and prompt intervention. Children with restrictive eating may be at heightened risk. Multicenter pediatric studies are required to further investigate the prevalence, causes, and natural history of malnutrition‐related cardiomyopathy.

## CONFLICT OF INTEREST STATEMENT

The authors declare no conflicts of interest.

## ETHICS STATEMENT

This case report was conducted in compliance with the ethical principles of the Declaration of Helsinki. Verbal informed consent for the publication of this case report was obtained from the patient's father, with the consent witnessed by a member of the clinical team. This approach was conducted with the utmost care to ensure the patient's confidentiality and respect for their rights.

## Supporting information


**Supplemental Figure 1**: Nutritional provision progression with parenteral and enteral nutrition. Initial estimated needs were calculated with WHO equation for basal metabolic rate using activity factor of 0.7‐1.2 gave a goal of 660‐1000 kcal/day. Based on this 1‐2 g/kg/day of amino acid were the goal for his age. Started at 0.5 g/kg per day of amino acid when initiated on parenteral nutrition due to his severe protein‐energy malnutrition and high risk of refeeding. PN = parenteral nutrition.
