# Supplementary figures and images for: Malnutrition‐related cardiomyopathy in a pediatric patient with autism spectrum disorder
Source: JPGN Rep. 2024 Oct 25;6(1):39–42. doi: 10.1002/jpr3.12142 (PMC11810803; doi:10.1002/jpr3.12142)

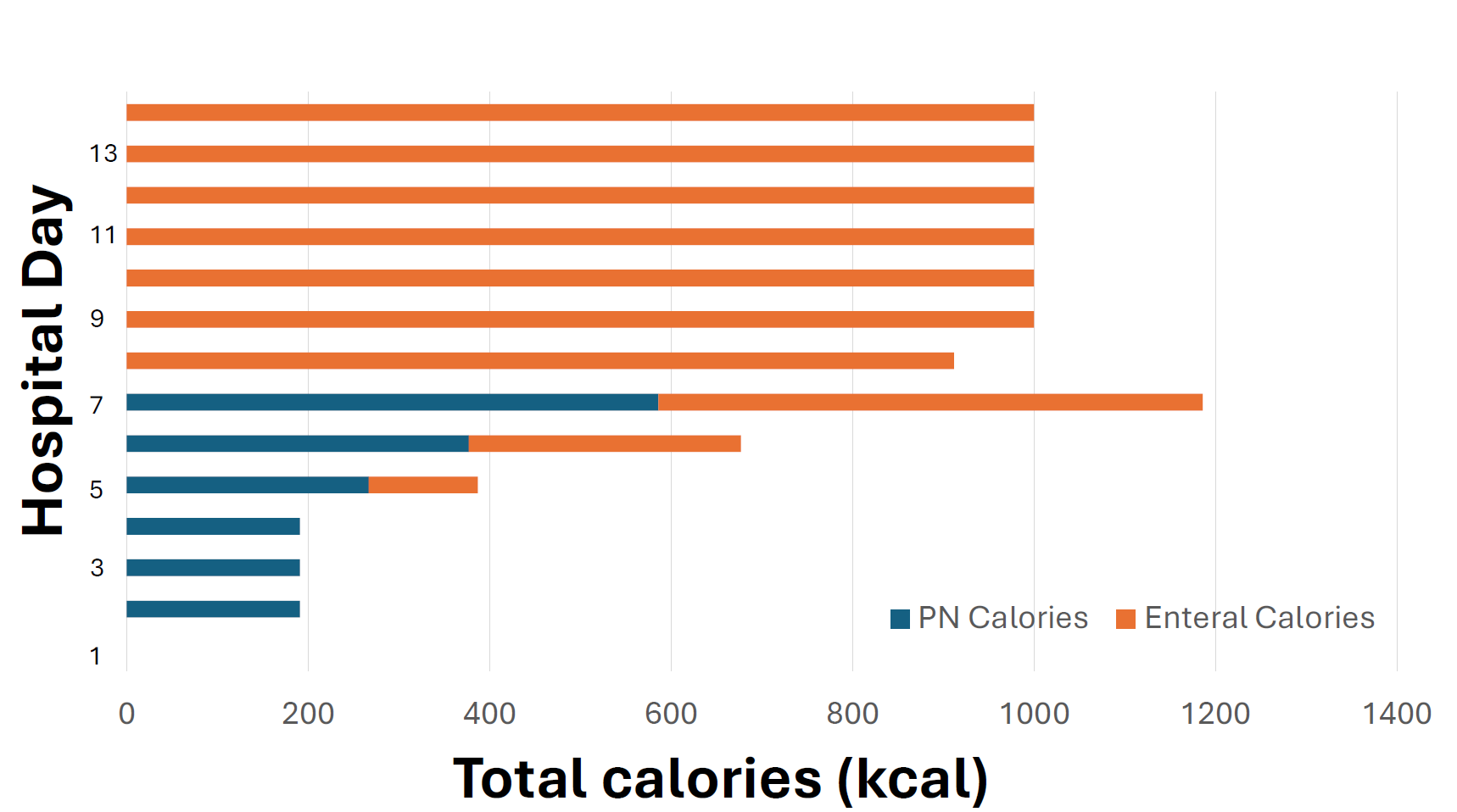

Supplement: Supplementary file 1 — Supplemental Figure 1: Nutritional provision progression with parenteral and enteral nutrition. Initial estimated needs were calculated with WHO equation for basal metabolic rate using activity factor of 0.7‐1.2 gave a goal of 660‐1000 kcal/day. Based on this 1‐2 g/kg/day of amino acid were the goal for his age. Started at 0.5 g/kg per day of amino acid when initiated on parenteral nutrition due to his severe protein‐energy malnutrition and high risk of refeeding. PN = parenteral nutrition. [file JPR3-6-39-s001.docx]
